# Clinical *Streptococcus pneumoniae* isolates induce differing CXCL8 responses from human nasopharyngeal epithelial cells which are reduced by liposomes

**DOI:** 10.1186/s12866-016-0777-5

**Published:** 2016-07-19

**Authors:** Denja Baumgartner, Suzanne Aebi, Denis Grandgirard, Stephen L. Leib, Annette Draeger, Eduard Babiychuk, Lucy J. Hathaway

**Affiliations:** Faculty of Medicine, Institute for Infectious Diseases, University of Bern, Friedbühlstrasse 51, Bern, CH-3001 Switzerland; Faculty of Medicine, Institute of Anatomy, University of Bern, Baltzerstrasse 2, Bern, CH-3012 Switzerland

**Keywords:** *Streptococcus pneumoniae*, CXCL8, Liposomes, Pneumolysin, Capsule

## Abstract

**Background:**

*Streptococcus pneumoniae* causes several human diseases, including pneumonia and meningitis, in which pathology is associated with an excessive inflammatory response. A major inducer of this response is the cholesterol dependent pneumococcal toxin, pneumolysin. Here, we measured the amount of inflammatory cytokine CXCL8 (interleukin (IL)-8) by ELISA released by human nasopharyngeal epithelial (Detroit 562) cells as inflammatory response to a 24 h exposure to different pneumococcal strains.

**Results:**

We found pneumolysin to be the major factor influencing the CXCL8 response. Cholesterol and sphingomyelin-containing liposomes designed to sequester pneumolysin were highly effective at reducing CXCL8 levels from epithelial cells exposed to different clinical pneumococcal isolates. These liposomes also reduced CXCL8 response from epithelial cells exposed to pneumolysin knock-out mutants of *S. pneumoniae* indicating that they also reduce the CXCL8-inducing effect of an unidentified pneumococcal virulence factor, in addition to pneumolysin.

**Conclusion:**

The results indicate the potential of liposomes in attenuating excessive inflammation as a future adjunctive treatment of pneumococcal diseases.

**Electronic supplementary material:**

The online version of this article (doi:10.1186/s12866-016-0777-5) contains supplementary material, which is available to authorized users.

## Background

*Streptococcus pneumoniae* is a major human pathogen responsible for diseases including pneumonia and meningitis which are characterized by significant inflammatory responses [1]. Such responses are associated with the release of cytokines including CXCL8, also known as interleukin (IL)-8 as well as IL-6, IL-1β, granulocyte-macrophage colony stimulating factor (GM_CSF), transforming growth factor (TGF) α and β [2]. Triggering of an inflammatory response may be due to interaction between host cells and several different bacterial factors. Respiratory epithelial cells express pattern-recognition receptors (PRRs) such as Toll-like receptors (TLRs) 2–6 with TLR2 recognizing bacterial factors such as peptidoglycan and lipoteichoic acid [3]. Epithelial cells expressing TLRs may release CXCL8 during respiratory infection which has chemotactic activity for neutrophils and monocytes [4]. Innate immunity is also comprised of antimicrobial effectors including antimicrobial peptides which have been shown to be effective against *S. pneumoniae* [5].

Excessive inflammation can have a deleterious effect on the host and therefore treatments are sought which can moderate this response to the bacteria. A major trigger for inflammatory cytokine release from the host is thought to be the cholesterol-dependent toxin, pneumolysin. However, natural variants of pneumolysin exist with different haemolytic activity, particularly a non-haemolytic pneumolysin associated with serotype 1 strains of multi-locus sequence type (MLST) ST306 [6, 7]. A liposomal formulation has been designed with the specific aim of sequestering pore-forming toxins, including pneumolysin, thereby preventing it from inserting into host cell membranes and initiating the inflammatory response [8]. These liposomes have so far been tested on a limited number of pneumococcal strains [8, 9].

As well as the virulence factor pneumolysin, most pneumococci express one of more than 90 different polysaccharide capsules [10]. The presence or absence of polysaccharide capsule can affect CXCL8 release by human respiratory epithelial cells in vitro [11] but whether the capsule serotype plays a role in CXCL8 induction is unknown. Here we have tested whether clinical isolates of different serotypes induced different levels of CXCL8 release from respiratory epithelial cells and whether this is linked to capsule type using mutants with the same genetic background expressing capsules of different serotypes.

To date, studies on the effectiveness of liposomes have been confined to a limited number of pneumococcal strains. Here we tested the effectiveness of liposomes against a panel of clinical isolates including serotype 1 strains of different haemolytic activity. Pneumolysin mutants were also tested to determine whether these liposomes had an effect on any other CXCL8-inducing bacterial factor.

## Methods

### Bacterial strains

Fourteen wild type *Streptococcus pneumoniae* strains with different serotypes and haemolytic activity were used, including strain 202.67, a non-haemolytic serotype 1 strain of ST306. Also used were one non-encapsulated mutant of strain 106.66 (named 106.66 Janus) and 8 capsule switch mutants of strain 106.66, for example mutant 106.66cpsB201.73 refers to strain 106.66 (originally serotype 6B) which has had its capsule operon replaced by that of strain B201.73 resulting in it becoming serotype 19 F [12].

Also, 3 mutants of the D39 strain in which the pneumolysin and/or capsule gene had been deleted were used. The D39 strain lacking pneumolysin (D39Δply) was a kind gift of Jeremy Brown, UCL, London. The bacteria are listed in Table 1 and mutant construction is described in previous publications [11, 12].

**Table 1:** Wild type and mutant *S. pneumoniae* strains used

Strain	Capsule serotype	Description
B201.73	19F	Wild types (clinical isolates) [12]
103.57	23F	
B101.77	14	
307.14	18C	
109.74	9V	
207.31	15	
106.66	6B	
208.41	7F	
P21	3	Wild type [13]
106.66 Janus	nonencapsulated	Capsule operon replaced by a Janus cassette [12]
106.66cps106.66	6B	Capsule switch mutants [12]
106.66cpsB201.73	19F	
106.66cps103.57	23F	
106.66cpsB101.77	14	
106.66cps307.14	18C	
106.66cps109.74	9V	
106.66cps207.31	15	
106.66cps208.41	7F	
B103.21	1	Wild type, non-haemolytic (current study)
211.25	1	Wild type, poorly haemolytic (current study)
207.06	1	Wild type, haemolytic (current study)
202.67	1	Wild type, non-haemolytic (current study)
D39	2	Wild type [11]
D39Δply	2	Mutant lacking pneumolysin [11]
D39Δcps	nonencapsulated	Mutant lacking capsule [11]
D39ΔplyΔcps	nonencapsulated	Mutant lacking pneumolysin and capsule [11]

### Bacterial culture

Bacterial stocks were stored at −80 °C using Protect bacterial preservers (Technical Service Consultants, Heywood, U.K.). The bacteria were plated out on Columbia sheep blood agar (CSBA) plates and incubated overnight at 37 °C at 5 % CO_2_. Three to ten colonies were used to inoculate 5 ml Brain Heart Infusion (BHI) broth (Becton Dickinson and Company, le Pont de Claix, France) for overnight culture in a waterbath at 37 °C. 1 ml of the overnight culture was added to 9 ml BHI and incubated in a waterbath at 37 °C until reaching mid-log phase, OD_600nm_ = 0.4. The bacterial cells were collected by centrifugation of 10 ml culture and were washed with Minimum Essential Media (MEM, Gibco, Life Technologies, Switzerland). The pellets were re-suspended in 10 ml MEM.

### Detroit cell culture

The human pharyngeal epithelial cell line Detroit 562 (ATCC CCL-138) was cultured submerged in complete medium consisting of Minimum Essential Media (MEM) with 10 % heat-inactivated fetal calf serum (FCS), 2 mM of L-glutamine, 0.075 % sodium bicarbonate, 1x MEM non-essential amino acid solution, 1 mM sodium pyruvate, 100 μg/ml streptomycin and 100 U/ml penicillin (all from Gibco, Life Technologies, Switzerland) at 37 °C at 5 % CO_2_. Cells were harvested using 0.05 % Trypsin-EDTA (Gibco, Switzerland) when the cells reached 70–90 % confluence.

### CXCL8 (IL-8) cytokine assay

3 × 10^5^ Detroit cells in 1 ml MEM without antibiotics, was added to each well of a 24-well plate (TPP tissue culture plates, Sigma-Aldrich,). The plate was incubated overnight at 37 °C at 5 % CO_2_ then integrity of the monolayer checked by microscopy. The medium was aspirated and 0.5 ml MEM without FCS or antibiotics added per well.

A suspension of bacteria of approximately 6 × 10^6^ CFU/ml was made (to give an estimated MOI of 10). Serial dilutions of the suspension were plated out for accurate quantification of CFU/ml, and therefore MOI.

Liposomes (CAL02) were provided by LASCCO (Geneva, Switzerland) and have previously been shown to be neither bactericidal nor toxic to epithelial cells [8] (Additional file 1: Figure S2). The liposome concentrations utilized in these experiments were chosen based preliminary experiments using liposome concentration ranging from 50ug to 1 mg (data not shown). Therefore, 1 mg or 100 μg of liposomes were added per well followed by 0.5 ml MEM containing the bacteria (6 × 10^6^ CFU/ml) in the subsequent experiments The plate was centrifuged at 120 x g for 3 min at 25 °C and then incubated at 37 °C at 5 % CO_2_. For experiments involving lysis of the P21 strain of bacteria by antibiotic, 10 μl / well of a 10 mg/ml solution of ceftriaxone (Rocephine®, Roche Pharma, Basel, Switzerland) was added after 3 h of incubation. After incubating for a total of 24 h at 37 °C at 5 % CO_2_ the supernatant was collected in 1.5 ml tubes, spun down at 20 000 x g for 3 min at room temperature and the supernatant stored at −80 °C. CXCL8 concentrations were measured by ELISA (R&D systems ELISA kits, Abingdon, United Kingdom). Experiments were performed in triplicate on three different days. Mean CXCL8 concentration for Detroit cells plus 1 mg liposomes, but no bacteria, for all experiments involving liposomes was 412 pg/ml, indicating that the liposomes do not have a cytotoxic effect on eukaryotic cells as reported previously [8].

### Haemolysis assay

The bacteria were grown overnight on CSBA plates at 37 °C at 5 % CO_2_ and then cultured overnight in 5 ml BHI containing 5 % FCS. 1 ml of overnight culture was added to 5 ml BHI + FCS and subcultured until OD_600nm_ 0.4. The bacteria were then centrifuged at 5000 x g for 10 min at room temperature and resuspended in 100 μl PBS. To release haemolysins from the bacteria, they were sonicated for 5 min on ice. In a round-bottomed 96-well plate (Sarstedt, NC, USA), 50 μl PBS (pH 7.4) was added per well along with 50 μl of bacterial sonicate or 50 μl pneumolysin (2 mg/ml, as a positive control) and doubling dilutions made across the plate. One row of wells was used as negative control (PBS only). 50 μl of 2 % sheep red blood cell suspension in PBS was added per well and incubated for 30 min at 37 °C and lysis monitored.

### Statistics

To assess the significance of the results ANOVA (with Tukey’s post hoc test) or student *t* test was used in GraphPad Prism as indicated. A p value < 0.05 was considered statistically significant.

## Results

### Clinical pneumococcal isolates varied in their induction of CXCL8 from epithelial cells

**Fig. 1:**
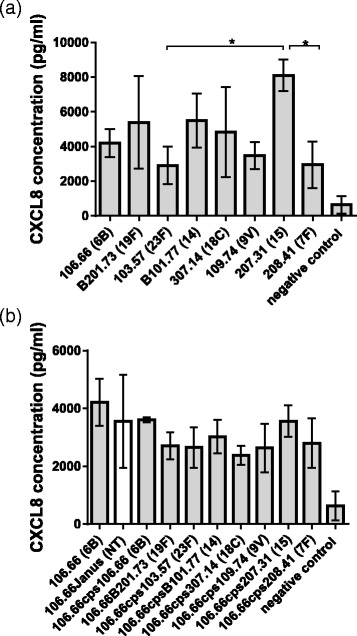
Effect of pneumococcal strains of different serotypes on CXCL8 induction in human nasopharyngeal epithelial cells. Detroit 562 nasopharyngeal epithelial cells were assessed for CXCL8 release after exposure to wildtype (**a**) or capsule switch mutant (**b**) pneumococcal strains. **a** Strain 207.31 induced more CXCL8 than either strain 103.57 or strain 208.41 but there were no other significant differences in CXCL8 induction between the clinical isolates. **b** No significant differences in CXCL8 induction were detected between any of the strains of different serotypes but the same genetic background. All experiments were performed in triplicate. Serotypes are indicated in brackets, NT refers to the nontypeable capsule deletion mutant. Negative control refers to Detroit cells in the absence of bacteria. * indicates significant difference by ANOVA with Tukey’s post hoc test. Error bars indicate standard deviation

The CXCL8 response from Detroit 562 human pharyngeal epithelial cells was measured following exposure to a selection of clinical pneumococcal isolates of different serotypes. CXCL8 concentrations varied according to the pneumococcal strain, ranging from 2909 pg/ml for the serotype 23 F strain to 8092 pg/ml for the serotype 15 strain (Fig. 1a). (For all strains the value was significantly greater than the mean CXCL8 concentration for Detroit cells only which was 471 pg/ml). The strain of serotype 15 induced significantly more CXCL8 than either the strains of serotype 23 F or 7 F (assessed by ANOVA with Tukey’s post hoc test) but there were no other significant differences in CXCL8 induction between the clinical isolates. To determine whether serotype plays a role in level of CXCL8 response, we compared the effects of a 6B clinical isolate (strain 106.66) with its capsule deletion mutant (106.66Janus) and capsule switch mutants with the same genetic background expressing capsules of eight different serotypes (Fig. 1b) i.e. with the genetic background of strain 106.66 but expressing the capsules of the clinical strains in Fig. 1a (Table 1). CXCL8 response varied between the lowest value of 2384 pg/ml observed for serotype 18C and the highest 4209 pg/ml for the 6B parent strain but no differences were statistically significant by ANOVA.

### Liposomes reduced CXCL8 from epithelial cells exposed to different clinical pneumococcal isolates

**Fig. 2:**
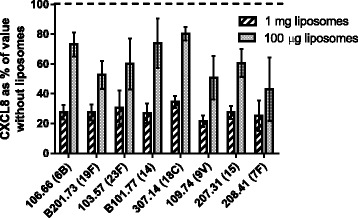
Liposomes (1 mg or 100 μg) reduced CXCL8 concentration from human nasopharyngeal epithelial cells induced by different pneumococcal strains. Values shown are means of three independent experiments and are the values with 1 mg or 100 μg of liposomes expressed as percentages of the CXCL8 concentrations obtained in the absence of liposomes in the presence of wild type clinical isolates. Liposomes reduced the CXCL8 response to all pneumococcal strains tested. Numbers in brackets indicate serotypes. Error bars indicate standard deviation

As the most important virulence factor in inducing a CXCL8 response in the epithelial cells is thought to be pneumolysin, we tested the effectiveness of liposomes which absorb cholesterol-dependent toxins, such as pneumolysin, in reducing the CXCL8 response to different clinical pneumococcal isolates. We found that these liposomes were effective for all the pneumococcal isolates tested (Fig. 2). 1 mg of liposomes reduced the concentration of CXCL8 to a mean value of 27.7 % of that in the absence of liposomes. 100 μg reduced CXCL8 levels to a mean of 61.8 % of those in the absence of liposomes. (i.e. 1 mg of liposomes reduced CXCL8 levels by a mean of 72.3 % and 100 ug by 38.2 %).

### Lytic antibiotic is required to induce CXCL8 response to serotype 3 strain P21, which was reduced by liposomes

**Fig. 3:**
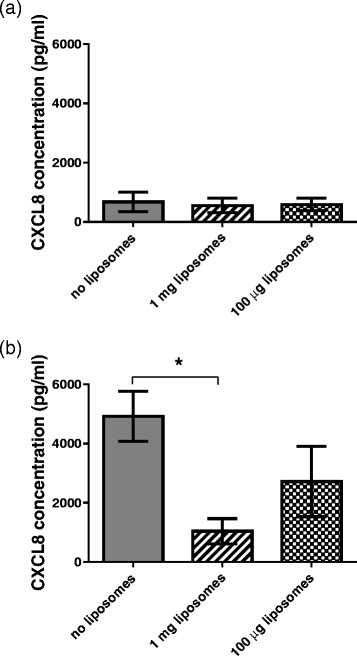
Treatment of serotype 3 strain P21 with lytic antibiotic is required for release of CXCL8 from human nasopharyngeal epithelial cells which is reduced by liposomes. Values shown are means of three independent experiments and are the absolute CXCL8 concentration in the (**a**) absence of antibiotic and (**b**) following addition of the lytic antibiotic ceftriaxone in the absence or presence of 1 mg or 100 μg of liposomes. Lytic antibiotic caused strain P21 to induce CXCL8 from the epithelial cells, the level of which was reduced by liposomes. * indicates significant difference. Error bars indicate standard deviation

Unlike the other wild type clinical isolates in this study, strain P21, serotype 3, has been passaged in an animal model and has an extremely thick capsule. Using the same protocol as used for the strains in Figs. 1 and 2 we found no induction of CXCL8 release by strain P21 (Fig. 3a). However, treatment with the lytic antibiotic ceftriaxone enabled strain P21 to induce a CXCL8 response which was reduced by the presence of liposomes (Fig. 3b).

### Reduction of CXCL8 response by liposomes depended on haemolytic activity of serotype 1 pneumococcal strains

**Fig. 4:**
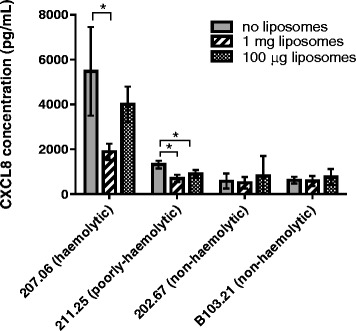
CXCL8 responses of human nasopharyngeal epithelial cells to serotype 1 pneumococcal clinical isolates with different haemolytic activities in the absence and presence (1 mg or 100 μg) of liposomes. For the haemolytic and poorly haemolytic strains, liposomes significantly reduced CXCL8 concentration (*p* < 0.05 for 1 mg liposomes). The non-haemolytic strains induced so little CXCL8 that no reduction was observable by adding liposomes. * indicates significant difference. Error bars indicate standard deviation

Haemolytic activity was determined for four serotype 1 clinical isolates (Additional file 1: Figure S1) and used to assign them as haemolytic, poorly haemolytic or non-haemolytic. The non-haemolytic strains did not trigger CXCL8 levels above the baseline secretion by epithelial cells suggesting that, without active pneumolysin, these strains were not releasing any other significant CXCL8-inducing factors. For the haemolytic and poorly haemolytic strains, liposomes significantly reduced CXCL8 concentration (*p* < 0.05 for 1 mg liposomes) (Fig. 4). Since the non-haemolytic strains did not induce CXCL8 production in Detroit 562 cells, liposome treatment had no observable effect.

### Liposomes acted on pneumolysin and another virulence factor

**Fig. 5:**
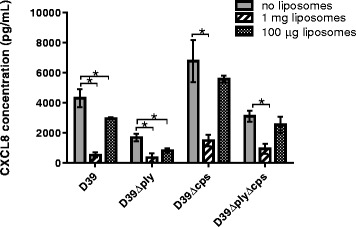
CXCL8 responses of human nasopharyngeal epithelial cells to pneumococcal strain D39 (serotype 2) and its mutants lacking pneumolysin (D39Δply) or capsule (D39Δcps) or both (D39ΔplyΔcps) in the absence and presence (1 mg or 100 μg) of liposomes. Liposomes reduced CXCL8 levels following exposure to the wildtype strain D39 strain but also reduced the CXCL8 response to its mutants lacking pneumolysin. * indicates significant difference. Error bars indicate standard deviation

To determine whether the effect of the liposomal formulation was entirely due to their ability to neutralize pneumolysin, we analysed their effect on CXCL8 levels from Detroit cells exposed to the pneumococcal laboratory strain D39 (serotype 2) and its mutants in which pneumolysin or capsule or both had been deleted. Figure 5 shows that, as expected, liposomes reduced CXCL8 levels following exposure to D39 strain, which possesses both capsule and pneumolysin. However, the liposomes also had a significant effect in the mutant in which the pneumolysin gene had been deleted (D39Δply) indicating efficacy against a proinflammatory bacterial factor other than pneumolysin. Consistent with this, 1 mg liposomes also reduced CXCL8 concentration induced by the capsule-deficient mutant (D39Δcps) (which has pneumolysin) and the mutant lacking both pneumolysin and capsule (D39ΔplyΔcps).

## Discussion

An excessive inflammatory response can cause damage to the host during pneumococcal diseases which may be exacerbated by treatment with lytic antibiotics as they cause release of inflammatory factors. The cholesterol-dependent toxin pneumolysin plays an important role in induction of inflammation but the presence of capsule has also been shown to have an influence [11, 14, 15]. Here we determined whether the inflammatory response, as measured by release of the inflammatory cytokine CXCL8 from human nasopharyngeal epithelial cells in vitro, was affected by the pneumococcal serotype. The pneumococcal clinical isolates tested induced different amounts of the inflammatory cytokine CXCL8 (Fig. 1a). By using capsule switch mutants we have shown that capsule serotype had no significant effect on CXCL8 levels, under the in vitro conditions used (Fig. 1b). Deletion of capsule in strain D39 resulted in an increase in release of CXCL8 from the epithelial cells but when capsule was deleted in strain 106.66 no significant difference in CXCL8 was observed, suggesting a strain-specific effect. Cytokine networks involved in pneumococcal infections are incompletely understood but IL-1β has been shown to regulate CXCL8 release from epithelial cells in reponse to *S. pneumoniae* [16] and would be an interesting target for future study. Furthermore, it has also been shown, in vivo, that pneumococcal capsule could impair recognition by the innate immune system, in particular the Toll-like receptor mediated pathways [17]. However, the most evident difference was between wild types and pneumolysin mutants indicating that pneumolysin is the predominant CXCL8 inducer.

Liposomes designed to sequester cholesterol-dependent toxins such as pneumolysin [8] caused a marked, and dose dependent, decrease in CXCL8 release from the epithelial cells for all the serotypes of clinical isolates tested (Fig. 2). This indicates the potential of this liposome treatment to be effective against pneumococcal diseases caused by different pneumococcal strains in patients.

In pneumococcal meningitis mortality and morbidity are exacerbated by an excessive inflammatory response. Treatment with lytic antibiotic, although effective at killing the bacteria, causes release of pneumolysin triggering an even greater inflammatory response. In an infant rat model of pneumococcal meningitis a serotype 3 strain (P21) is often used to induce the disease [18] and here we have shown that in vitro, following antibiotic lysis, which would release the pneumolysin, liposome treatment greatly reduced CXCL8 levels from the epithelial cells (Fig. 3). This raises the possibility of a future anti-inflammatory treatment for pneumococcal meningitis including this liposomal formulation. Serotype 3 strains are also associated with severe forms of pneumonia and with septic shock [19, 20] and so the effectiveness of the liposome treatment against this serotype, as least in vitro, is encouraging.

In nature several different alleles of pneumolysin exist with differing haemolytic activity [6, 21]. Serotype 1 has particularly been noted to include non-haemolytic and poorly-haemolytic variants. We found liposomes to be effective at reducing the CXCL8 response to haemolytic and poorly-haemolytic strains (Fig. 4). Non-haemolytic strains did not trigger CXCL8 levels above the baseline secretion by epithelial cells so liposomes did not reduce the level further. Overall, the results indicate that liposomes may be useful in counteracting the inflammatory effect of a wide range of clinical pneumococcal strains of different haemolytic activity as well as different serotypes.

Using pneumolysin and capsule mutants of strain D39 (Fig. 5), we showed for the first time that the liposomes act not only on pneumolysin, but also significantly reduce the CXCL8 response to another factor which remains to be identified.

## Conclusions

We found that clinical pneumococcal isolates varied in their capacity to induce CXCL8 production by respiratory epithelial cells. Capsule type had little effect on this variation compared with pneumolysin which had the predominant effect. Liposomes designed to sequester pneumolysin were extremely effective in vitro in reducing CXCL8 levels from epithelial cells exposed to a range of clinical pneumococcal isolates. Liposomes also reduced CXCL8 release induced by mutant pneumococci lacking pneumolysin indicating that they also act on another factor.

## Additional file

Additional file 1: Figure S1. Results of haemolysis assay for four different serotype 1 clinical isolates. **Figure S2.** Showing no effect on viability of pneumococcal strain 106.66 by 1mg of liposomes after 30, 120 and 240 minutes of culture. (DOC 439 kb)
