# Supplementary material for: Clinical Streptococcus pneumoniae isolates induce differing CXCL8 responses from human nasopharyngeal epithelial cells which are reduced by liposomes
Source: BMC Microbiol. 2016 Jul 19;16:154. doi: 10.1186/s12866-016-0777-5 (PMC4950757; doi:10.1186/s12866-016-0777-5)
Supplement: Additional file 1: Figure S1. — Results of haemolysis assay for four different serotype 1 clinical isolates. Figure S2. Showing no effect on viability of pneumococcal strain 106.66 by 1mg of liposomes after 30, 120 and 240 minutes of culture. (DOC 439 kb) [file 12866_2016_777_MOESM1_ESM.doc]

**
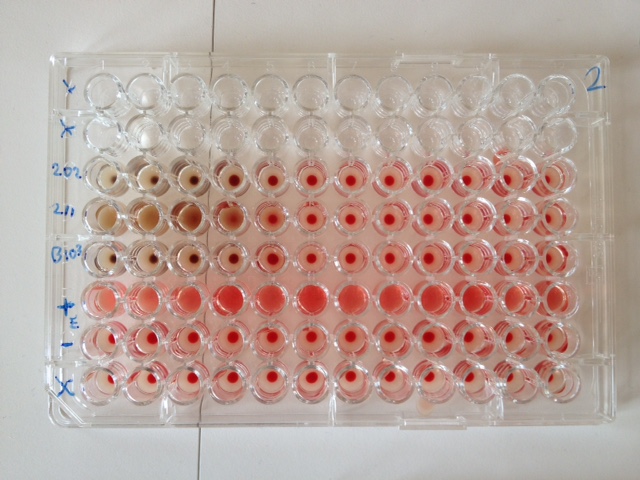
**

202.67 non-haemolytic

211.25 poorly-haemolytic

B103.21 non-haemolytic

+ (pneumolysin)

- (PBS)

207.06 haemolytic

**
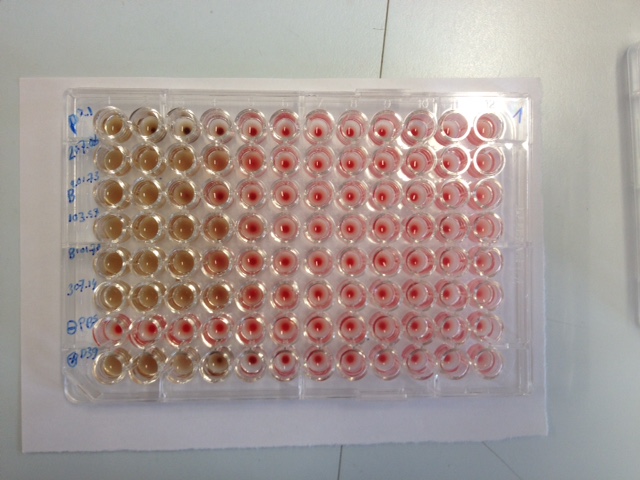
**

**Supplementary Figure S1 – Results of haemolysis assay for four different serotype 1 clinical isolates.** 50 µl PBS (pH 7.4) was added per well along with 50 µl of bacterial sonicate or 50 µl pneumolysin (2 mg/ml, as a positive control) to the wells in the first column and doubling dilutions made across the plate from left to right. One row of wells was used as negative control (PBS only). 50 µl of 2% sheep red blood cell suspension in PBS was added per well and incubated for 30 minutes at 37°C and then lysis was observed.

**Supplementary Figure S2 – Showing no effect on viability of pneumococcal strain 106.66 by 1mg of liposomes after 30, 120 and 240 minutes of culture.**
